# Inadequate energy and protein intake, underweight and malnutrition are associated with in‐hospital mortality among COVID‐19 rehabilitation patients during the omicron outbreak in Hong Kong

**DOI:** 10.1002/agm2.12220

**Published:** 2022-08-23

**Authors:** Terry Ho Yan Ting, Tiffany Hoi Man Lo, Winnie Wing Tung Lo, Qi Ding, Daniel Ka Lok Yuk, Elsie Hui, Maria Wing Sze Tang

**Affiliations:** ^1^ Dietetics Department Shatin Hospital Hong Kong China; ^2^ Department of Medicine and Geriatrics Shatin Hospital Hong Kong China

**Keywords:** COVID‐19 patient, malnutrition, mortality

## Abstract

**Objective:**

Malnourished COVID‐19 patients were prone to higher mortality and longer length of stay (LOS). This study aims to investigate the malnutrition risk prevalence in the COVID‐19 patients and how other nutritional indicators are related to the clinical outcomes in a rehabilitation hospital.

**Methods:**

A retrospective cross‐sectional study involved 174 COVID‐19 patients during the rehabilitation phase. Malnutrition risk, nutritional indicators, mortality, and LOS were compared among different risk groups. Albumin, nutrition intake, and body mass index (BMI) were investigated for their effects on the clinical outcomes.

**Results:**

The prevalence of malnutrition risk was 94.9%; those older were higher in malnutrition risk. BMI, energy and protein intakes decreased as the malnutrition risk increased. Albumin, energy and protein intakes were lower in the death group. The high malnutrition risk group and severely underweight patients had 2.7 times and 2.2 times higher in‐hospital death, respectively. For subjects ≥75 years old, the odds ratio to death was 6.2 compared to those <75 years old.

**Conclusion:**

We observed a high malnutrition risk of 94.9% in COVID‐19 patients. Patients with malnutrition risk had a lower BMI, lower nutritional intake, and a higher chance of in‐hospital death. These results reinforced the importance of nutrition management in COVID‐19 patients.

## Graphical abstract

One hundred seventy‐four COVID‐19 patients were retrieved for analysis. The prevalence of malnutrition risk was 94.9%; those older were higher in malnutrition risk. BMI, energy and protein intake decreased as the malnutrition risk increased. Albumin, energy and protein intakes were lower in the death group. The high malnutrition risk group and severely underweight patients had 2.7 times and 2.2 times higher to in‐hospital death, respectively. In subjects ≥75 years old, the odds ratio to death is 6.2 compared to those <75 years old.

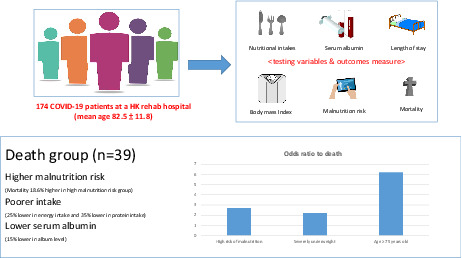

## 1 INTRODUCTION

Highly infectious severe acute respiratory syndrome coronavirus 2 (SARS‐CoV‐2) first emerged in humans at the end of 2019.^1^ However, Omicron, a new variant of SARS‐CoV‐2, had spread rapidly in Hong Kong by January 2022 and infected around 3.6 million people by 14 March 2022.^2^, ^3^ The coronavirus disease 2019 (COVID‐19) death rate in Hong Kong was reported in March 2022 as 25 per 100,000 residents, and more than 95% of the dying patients were elderly and not fully vaccinated.^4^ The average hospital length of stay (LOS) in Hong Kong in 2020 was reported to be 5.17 days.^5^ The elderly and those with comorbidities, such as diabetes, cardiovascular disease, cancer, and renal disease, are more prone to severe complications and mortality.^6^ A study that reviewed data from 3665 COVID‐19 patients in China suggested that mortality and the admission rate both increase with age.^7^ Other factors, such as nutritional status, are suggested to be closely related to the patients' clinical outcomes, namely mortality and LOS.^8^, ^9^

It is well‐documented that patients with a poor nutritional status are prone to poorer clinical outcomes, including higher in‐hospital mortality and longer LOS.^10^, ^11^ Malnutrition is defined as a state of excess or deficient nutrition, in terms of macro‐ and micronutrients, that causes adverse clinical, functional, and economic outcomes.^12^ Hospitalized patients have a high malnutrition rate, ranging from 9.2% to 77.8%, and malnourished patients stay longer in hospital and have a higher mortality rate.^12^, ^13^, ^14^ Malnutrition is frequently seen in COVID‐19 patients, and studies have suggested that the malnutrition rate in COVID‐19 patients ranges from 27.5% to 82.6% and that malnourished individuals are more prone to in‐hospital death compared to well‐nourished patients.^15^, ^16^, ^17^, ^18^, ^19^

Malnutrition is strongly associated with muscle loss in both community‐dwelling and hospitalized individuals.^20^, ^21^ Weight loss is common in COVID‐19 patients, and around 35% of patients have >5% unintentional weight loss.^22^ Inflammation, gastrointestinal tract symptoms, disease‐associated reductions in food intake, loss of appetite/taste, and immobilization are some of the major factors that contribute to the unintentional weight loss in COVID‐19 patients.^15^, ^16^, ^23^, ^24^, ^25^ Being underweight is a predictor of increased in‐hospital mortality.^26^ Studies have suggested that COVID‐19 patients with a body mass index (BMI) under 18.5 kg/m^2^ stay longer in hospital and have a higher mortality rate.^27^, ^28^, ^48^

Inadequate energy and protein intakes also contribute to the poor clinical outcome of COVID‐19 patients, and in the hospitalized patients, the intakes were not adequate.^13^, ^29^ The European Society for Clinical Nutrition and Metabolism suggested providing an energy level of 27–30 kcal/kg body weight and a protein intake of ≥1 g/kg body weight to COVID‐19 patients in 2020.^30^ A study involving 52 intensive care unit (ICU) patients who had a protein intake of <0.8 g/kg body weight showed a higher mortality rate in these patients.^31^ Another study of 112 critically ill patients with energy deficits in the first week of admission also resulted in a higher mortality rate.^32^ Serum albumin has long been used to predict mortality and LOS.^33^ Studies have suggested that COVID‐19‐induced cytokine causes critical hypoalbuminemia and that patients with lower serum albumin have poorer survival rates and longer LOS.^34^, ^35^, ^36^

This study aims to determine the prevalence of malnutrition risk in a group of COVID‐19 rehabilitation adult inpatients admitted during a pandemic wave dominated by the Omicron BA.2 variant and the relationship of other nutritional risk factors to the hospital LOS and in‐hospital mortality in a rehabilitation and convalescent hospital in Hong Kong.

## 2 METHODS

This is a retrospective cross‐sectional study for service evaluation. The study protocol complied with the Declaration of Helsinki and was approved by the Joint Chinese University Hong Kong‐New Territories East Cluster Clinical Research Ethics Committee (CREC 2022.300). The study hospital is a 600‐bed rehabilitation and convalescent hospital serving the Hong Kong New Territories East Cluster. Adult patients with a confirmed diagnosis of COVID‐19 and with nutritional problems admitted to study hospital for rehabilitation were referred to a dietitian for assessment. Electronic dietetics records for patient assessment were stored in the clinical management system for record‐keeping purposes. Weight and height records were obtained and entered into the nursing electronic patient record system by the frontline ward staff during the patient admission procedure and BMI calculation was performed by the dietitian during the initial assessment. Baseline energy and protein intakes and the malnutrition risk level were estimated by the dietitian during the initial assessment for intervention purposes and entered into the electronic system for record keeping. Based on subjects' various nutrition markers such as food intake, output, weight change, BMI, blood test results, and clinical conditions, dietitians assigned a malnutrition risk level of low, medium, or high to the patient during each consultation. In addition, data on baseline serum albumin, LOS, and mortality were collected and entered into the dietetics electronic system by the patient's dietitian. One hundred seventy‐four records for COVID‐19 patients admitted to the study hospital for rehabilitation during the study period 25 February to 27 April 2022 were retrieved from the dietetics electronic record system for analysis. Hospital‐acquired COVID‐19 patients, nonrehabilitation patients, and patients who did not have baseline weight, height, energy and protein intake records were excluded from this review study.

Patients were categorized into three different malnutrition risk groups for descriptive analysis. Malnutrition risk prevalence was reported based on several variables, including age, BMI, serum albumin, energy and protein intakes, LOS, and mortality. Nutritional indicators, including malnutrition risk, BMI, energy and protein intake, and serum albumin, were then studied in both in‐hospital death and nondeath groups to establish their relationships. The risk of in‐hospital death was studied in the following groups: (1) high, medium, and low malnutrition risks; (2) severely underweight (BMI < 16.5 kg/m^2^) versus BMI ≥16.5 kg/m^2^; and (3) age ≥75 years versus <75 years. Descriptive statistics were obtained for all study variables, with categorical variables summarized as counts and percentages. One‐way analysis of variance (ANOVA), independent *t*‐test, and Pearson's chi‐square test was used to analyze the outcomes and compare means among the different malnutrition risk groups (malnutrition risk concerning BMI, energy and protein intakes, LOS, and mortality rate). A significance level of 5% (*p* ≤ 0.05) was used to indicate that a difference or relationship was considered statistically significant. Missing data were not imputed. The statistical software program IBM SPSS Statistics Package Version 26 was used to analyze the variables.

## 3 RESULTS

The mean age of the 174 subjects (74 females; 100 males) was 82.5 ± 11.8 years, ranging from 32 to 105 years. The overall prevalence of malnutrition risk in this group of COVID‐19 rehabilitation patients was 94.9% (medium risk: 77.0%; high risk: 17.9%; *p* = 0.021), and the older the patient, the higher the malnutrition risk (low risk: 72.3 ± 18.4 years; medium risk: 82.7 ± 11.3 years; high risk: 85.0 ± 10.5 years; *p* = 0.017). The BMI was significantly lower as the malnutrition risk increased (low risk: 21.0 ± 2.9 kg/m^2^; medium risk: 18.9 ± 3.7 kg/m^2^; high risk: 17.2 ± 3.0 kg/m^2^; *P* = 0.006). The mean daily energy and protein intakes were 749.7 ± 428.5 kcal and 32.9 ± 18.9 g, meeting 55.6% and 58.7% of the energy and protein requirements of the subjects, respectively. Both energy and protein intakes were significantly lower as the malnutrition risk increased (energy intake: low risk, 1233.9 ± 298.5 kcal; medium risk, 770.5 ± 408.5 kcal; high risk, 519.4 ± 410.0 kcal; *P* < 0.001; protein intake: low risk, 54.1 ± 16.0 g; medium risk, 33.5 ± 18.0 g; high risk, 24.2 ± 18.2 g; *P* < 0.001). The LOS was not significantly different among the three risk groups (Table 1).

**TABLE 1:** Demographic information, nutritional indicators and clinical outcome of COVID‐19 rehabilitation patients

Malnutrition risk					
	All patients (*n* = 174)	Low risk (*n* = 9; 5.1%)	Medium risk (*n* = 134; 77.0%)	High risk (*n* = 31; 17.9%)	*P*
Age (years)	82.5 ± 11.8	72.3 ± 18.4	82.7 ± 11.3	85.0 ± 10.5	0.017
Gender					
Male	100	7 (7%)	81 (81%)	12 (12%)	0.039
Female	74	2 (2.7%)	53 (71.7%)	19 (25.6%)	
Nutritional indicator					
BMI (kg/m^2^)	18.9 ± 3.7	21.0 ± 2.9	18.9 ± 3.7	17.2 ± 3.0	0.006
Energy intake (kcal)	749.7 ± 428.5	1233.9 ± 298.5	770.5 ± 408.5	519.4 ± 410.0	<0.001
Protein intake (g)	32.9 ± 18.9	54.1 ± 16.0	33.5 ± 18.0	24.2 ± 18.2	<0.001
Serum albumin (g/l)	25.0 ± 5.3	28.7 ± 6.2	25.1 ± 5.3	23.9 ± 5.1	0.061
Clinical outcome					
LOS (days)	19.3 ± 12.5	19.7 ± 5.9	19.7 ± 13.4	17.6 ± 10.7	0.710
Death	39	0	27 (69.2%)	12 (30.8%)	0.021

*Note*: Data are presented as the mean ± standard deviation (range).

Abbreviations: BMI, body mass index; LOS, length of stay.

There were 39 subjects who died during their admission, representing 22.4% of the sample population, with a mean LOS of 13.8 days ± 8.54 days, ranging from 2 days to 36 days. The in‐hospital mortality in the three risk groups was significantly different (low risk, 0; medium risk, 20.1%; high risk, 38.7%; *P* < 0.021). Other nutrition indicators, such as serum albumin and energy/protein intakes, were significantly lower for the in‐hospital death group (Table 1) compared to patients who survived: serum albumin (death group: 21.9 ± 4.4 g/L; survivor group: 26.0 ± 5.2 g/L; *p* < 0.001); energy intake (death group: 590.9 ± 486.9 kcal; survivor group: 795.6 ± 428.5 kcal; *P* = 0.004); and protein intake (death group: 26.4 ± 22.3 g; survivor group: 38.4 ± 17.5 g; *P* = 0.006). The BMI in the death and survivor groups was not significantly different (Table 2). When comparing the high malnutrition risk group to the low–medium risk group, the odds ratio for death was 2.7 (*P* = 0.006, 95% CI: 1.18–6.26). The odds ratio for death in the severely underweight group (BMI < 16.5 kg/m^2^) compared to those with BMI ≥16.5 kg/m^2^ was 2.2 (*p* = 0.034, 95% CI: 1.05–4.63). For subjects who were age ≥75 years, the odds ratio for death was 6.2 (*p* = 0.016, 95% CI: 1.42–27.22) compared to those age <75 years (Table 3).

**TABLE 2:** Nutritional factors and in‐hospital death of COVID‐19 rehabilitation patients

In‐hospital death				
	Yes (*n* = 39)	No (*n* = 135)	Overall	*P*
Malnutrition risk				
High	12 (38.7%)	19 (61.3%)	31	0.021
Medium	27 (20.1%)	107 (19.9%)	134	
Low	0	9 (100%)	9	
Serum albumin (g/l)	21.9 ± 4.4	26.0 ± 5.2	25.0 ± 5.3	<0.001
BMI (kg/m^2^)	17.9 ± 3.6	19.1 ± 3.6	18.9 ± 3.7	0.065
Energy intake (kcal)	590.9 ± 486.9	795.6 ± 428.5	749.7 ± 400.4	0.004
Protein intake (g)	26.4 ± 22.3	38.4 ± 17.5	33.0 ± 18.9	0.006

*Note*: Data are presented as the mean ± standard deviation (range).

Abbreviation: BMI, body mass index.

**TABLE 3:** Odds ratio for in‐hospital death

Odds ratio for in‐hospital death				
Comparison of variables		Odds ratio	*P* value	95% CI
High risk of malnutrition (*n* = 31)	Low–Medium risk of malnutrition (*n* = 143)	2.7	0.006	1.18–6.26
BMI < 16.5 kg/m^2^ (*n* = 52)	BMI ≥ 16.5 kg/m^2^ (*n* = 122)	2.2	0.034	1.05–4.63
Age ≥ 75 years (*n* = 138)	Age < 75 years (*n* = 36)	6.2	0.016	1.42–27.22

Abbreviations: BMI; Body Mass Index, CI; confidence intervals.

## 4 DISCUSSION

More than 6000 people died in Hong Kong due to the fifth wave of the Omicron outbreak from 31 December 2021 to 23 March 2022, and more than 95% of them were over 60 years old.^37^ This study was intended to provide evidence on the prevalence of malnutrition risk and the relationship between nutritional indicators and clinical outcomes among a group of COVID‐19 rehabilitation patients in Hong Kong during the outbreak dominated by the Omicron BA.2 variant. Hospital malnutrition is common in COVID‐19 patients, especially in the elderly, and their prognosis and clinical outcomes are worse than in young people.^15^, ^16^, ^17^, ^18^, ^19^, ^38^

Our study observed a malnutrition risk prevalence of 94.9%, higher than previous literature findings of 9.2%–77.8% from before the COVID‐19 outbreak.^12^, ^13^ This discrepancy might be explained by the etiology of COVID‐19, which negatively affects the gastrointestinal tract, immunity, and oral intake.^10^ It is well‐documented that malnourished COVID‐19 patients are more susceptible to in‐hospital death. This could be due to the disease‐associated hyperinflammation and immunosuppressive status.^8^, ^9^, ^42^, ^44^, ^45^ A systematic review suggested that the mortality risk in malnourished COVID‐19 patients was ten times higher than in well‐nourished patients.^10^ Another study on 348 subjects suggested that malnutrition was an independent predictor of in‐hospital mortality in patients with COVID‐19.^46^ The findings of this study revealed that 38.7% of patients died in the high malnutrition risk group compared to 20.1% and 0% in the medium and low‐risk groups, respectively. The in‐hospital mortality of patients with a high malnutrition risk was 2.7‐fold higher than in the medium–low‐risk group.

Recent evidence has shown that malnutrition is frequently seen in hospitalized elderly.^15^, ^16^, ^17^, ^18^, ^19^ Our study showed that hospital malnutrition risk increased with age among this group of COVID‐19 rehabilitation patients. With middle age defined as ≥75 years,^49^ we compared patients aged <75 years to middle‐aged or older patients and found that their risk of in‐hospital death was 6.2‐fold higher. This finding was aligned with a few recent COVID‐19 studies regarding the relationship of age to mortality: the older the patients, the higher the risk of in‐hospital death.^7^, ^50^

Underweight hospitalized patients were more prone to malnutrition and had poorer clinical outcomes than well‐nourished individuals.^39^ According to the World Health Organization, the normal BMI range for Asians is 18.5–23.0 kg/m^2^.^40^ Although the mean BMI of the patients was within the normal range, the high‐risk patients had a BMI of <18.5 kg/m^2^. Severe underweight is defined as BMI < 16.5 kg/m^2^.^47^ A recent study revealed that underweight COVID‐19 patients had a 1.44‐fold higher chance of death.^48^ Our study had the similar finding that the severely underweight patients (BMI < 16.5 kg/m^2^) had a 2.2‐fold higher chance of in‐hospital death than those with BMI ≥16.5 kg/m^2^.

Nutritional intake is an essential indicator of a patient's nutritional status. A study on patients with chronic obstructive pulmonary disease identified that those at risk of malnutrition were consuming 59% and 64% of the estimated requirements for energy and protein, respectively.^13^ Research on energy and protein intake in COVID‐19 patients is limited. Still, evidence has shown that hospitalized patients were not consuming adequate energy and protein and that poor nutritional intake is related to poor clinical outcomes.^13^, ^19^, ^41^ The European Society for Clinical Nutrition and Metabolism recommended providing 27–30 kcal/kg body weight and at least 1.0 g protein/kg body weight to COVID‐19 patients to maintain their nutritional needs.^30^ Our study suggested that COVID‐19 patients at higher malnutrition risk consume less energy and protein than those in the lower‐risk groups. In addition to reporting the exact amounts of energy and protein intake in this study, we also provide an overall adequacy level of energy and protein intake, which is rarely found in the currently available literature. Furthermore, our study found that subjects in the in‐hospital death group consumed less energy and protein than those in the survival group. However, the study of hospitalized patients' energy and protein intake is limited.

A few recent studies on COVID‐19 patients have reported that malnourished patients have a longer LOS than well‐nourished individuals.^10^, ^11^, ^42^ However, our study finding on the LOS and malnutrition risk was not aligned with these reports, and we did not observe any significant difference among the three malnutrition risk groups. This discrepancy can be explained by the disadvantage of a retrospective study with a convenience sampling method in which confounding factors other than clinical conditions, such as discharge issues, placement issues, and environmental/social factors, may affect the study's outcome. Furthermore, the limited number of cases with low malnutrition and the unequal samples could negatively affect the statistical power and increase the chance of Type II error.^43^

Our study showed that serum albumin was associated with mortality. The in‐hospital death group had a lower baseline serum albumin level than the survival group. This finding was aligned with current studies regarding the association of low serum albumin levels with an increasing risk of in‐hospital death in COVID‐19 patients.^34^, ^35^

One limitation of this study is that the patient population was confined to those admitted for rehabilitation; therefore, the findings may not be generalized to other specialty patients in other acute hospitals. In addition, as a retrospective cross‐sectional study with a convenience sampling method, the clinical outcome can be subject to confounding factors such as disease severity or the presence of comorbidities but also other operational/environmental factors (e.g., discharge planning and placement arrangement) due primarily to the severe impact of the Omicron outbreak on the local public healthcare system.

## 5 CONCLUSION

We observed a high malnutrition risk prevalence of 94.9% in COVID‐19 patients in a hospital rehabilitation setting. Patients at risk of malnutrition had a lower BMI, lower nutritional intake, and a higher chance of in‐hospital death. We also established that other nutritional indicators were associated with in‐hospital mortality. Patients who died during the same hospital admission had lower BMI, poorer nutritional intake, and were older. These results reinforced recent mass research studies on the importance of nutrition management in COVID‐19 patients and their clinical outcomes.^8^, ^9^, ^10^, ^11^, ^12^, ^15^, ^16^, ^17^, ^18^, ^19^, ^20^, ^21^, ^22^ Further research is recommended to investigate the effect of nutritional intervention on longer‐term prognosis in COVID‐19 patients.

## AUTHOR CONTRIBUTIONS

HMT Lo and WTW Lo were responsible for the dietetic care of the patients, and Q Ding, KLD Yuk, E Hui, and WSM Tang were responsible for supervising the clinical work of the frontline staff and providing treatment care to the patients. All authors provided feedback on the final report. All authors reviewed and edited the final manuscript.

## FUNDING INFORMATION

None.

## CONFLICT OF INTEREST

None of the authors were involved in any financial interests connected to the work.

## ETHICS STATEMENT

This is a retrospective cross‐sectional study for service evaluation. The study protocol complied with the Declaration of Helsinki and was approved by the Joint Chinese University Hong Kong‐New Territories East Cluster Clinical Research Ethics Committee (CREC 2022.300).

[Correction added on September 1, 2022, after first online publication: "CREC 2022.300" has been added in the ethics statement]
